# Neurochemical Modifications in the Hippocampus, Cortex and Hypothalamus of Mice Exposed to Long-Term High-Fat Diet

**DOI:** 10.3389/fnins.2018.00985

**Published:** 2019-01-08

**Authors:** Blanca Lizarbe, Ana Francisca Soares, Sara Larsson, João M. N. Duarte

**Affiliations:** ^1^Laboratory for Functional and Metabolic Imaging, École Polytechnique Fédérale de Lausanne, Lausanne, Switzerland; ^2^Instituto de Investigaciones Biomédicas “Alberto Sols”, Consejo Superior de Investigaciones Científicas – Universidad Autónoma de Madrid, Madrid, Spain; ^3^Department of Experimental Medical Science, Faculty of Medicine, Lund University, Lund, Sweden; ^4^Wallenberg Centre for Molecular Medicine, Faculty of Medicine, Lund University, Lund, Sweden

**Keywords:** glucose, insulin, diabetes, brain metabolism, synaptic dysfunction, gliosis

## Abstract

Metabolic syndrome and diabetes impact brain function and metabolism. While it is well established that rodents exposed to diets rich in saturated fat develop brain dysfunction, contrasting results abound in the literature, likely as result of exposure to different high-fat diet (HFD) compositions and for varied periods of time. In the present study, we investigated alterations of hippocampal-dependent spatial memory by measuring Y-maze spontaneous alternation, metabolic profiles of the hippocampus, cortex and hypothalamus by ^1^H magnetic resonance spectroscopy (MRS), and levels of proteins specific to synaptic and glial compartments in mice exposed for 6 months to different amounts of fat (10, 45, or 60% of total energy intake). Increasing the dietary amount of fat from 10 to 45% or 60% resulted in obesity accompanied by increased leptin, fasting blood glucose and insulin, and reduced glucose tolerance. In comparison to controls (10%-fat), only mice fed the 60%-fat diet showed increased fed glycemia, as well as plasma corticosterone that has a major impact on brain function. HFD-induced metabolic profile modifications measured by ^1^H MRS were observed across the three brain areas in mice exposed to 60%- but not 45%-fat diet, while both HFD groups displayed impaired hippocampal-dependent memory. HFD also affected systems involved in neuro- or gliotransmission in the hippocampus. Namely, relative to controls, 60%-fat-fed mice showed reduced SNAP-25, PSD-95 and syntaxin-4 immunoreactivity, while 45%-fat-fed mice showed reduced gephyrin and syntaxin-4 immunoreactivity. For both HFD levels, reductions of the vesicular glutamate transporter vGlut1 and levels of the vesicular GABA transporter were observed in the hippocampus and hypothalamus, relative to controls. Immunoreactivity against GFAP and/or Iba-1 in the hypothalamus was higher in mice exposed to HFD than controls, suggesting occurrence of gliosis. We conclude that different levels of dietary fat result in distinct neurochemical alterations in the brain.

## Introduction

The ever-increasing life expectancy has led to a dramatic increase in the prevalence of age-associated disorders namely metabolic syndrome and type 2 diabetes (T2D), and neurodegenerative disorders, such as Alzheimer’s disease (AD). Notably, there is a growing body of epidemiological evidence suggesting that both metabolic syndrome and T2D increase the risk of developing age-related cognitive decline, mild cognitive impairment, vascular dementia, and AD (Frisardi et al., 2010; Duarte, 2015).

In addition to the many components of metabolic syndrome that may affect brain function, brain insulin resistance is a proposed mechanistic link between T2D and AD. Indeed, post-mortem analyses of brains from dementia patients revealed that insulin receptors are downregulated, and pointed toward a major role of neuronal insulin resistance in the etiology of AD (Steculorum et al., 2014; Duarte, 2015). Insulin receptors are widely distributed in the brain (Havrankova et al., 1978; Hill et al., 1986). Insulin and insulin-like growth factor 1 (IGF-1), which have converging signaling pathways in the brain, regulate the neuronal control of energy and glucose homeostasis by acting on hypothalamic nuclei (Steculorum et al., 2014), and also regulate cognitive processes through its actions on neurotransmission, synaptic plasticity, and neurogenesis within cortical and hippocampal circuits (Fernandez and Torres-Alemán, 2012).

A number of components of the metabolic syndrome such as hyperglycemia, microvascular complications, insulin resistance, dyslipidemia and hypertension occur in diabetes, and are risk factors for cognitive dysfunction (Duarte, 2015; Moheet et al., 2015). Although this combination of factors with potential impact on the central nervous system makes the mechanisms of diabetes-induced brain dysfunction difficult to study, research on different rodent models of diabetes have found that diabetic conditions lead to synaptic deterioration that results in defective neurotransmission and synaptic plasticity (Duarte et al., 2009, 2012a; Calvo-Ochoa et al., 2014), as well as neuroinflammation and astrogliosis (Saravia et al., 2002; Baydas et al., 2003; Duarte et al., 2009, 2012a; Calvo-Ochoa et al., 2014).

Changes in the cellular integrity of neurons and astrocytes are likely reflected on brain metabolic regulation, as well as metabolic profiles. Indeed, studies employing MRS, which allow to quantify brain metabolite profiles in a non-invasive manner, have generally identified reduced levels of the putative neuronal marker *N*-acetylaspartate, as well as increased in *myo*-inositol content (a proposed marker of astrocyte density) in brains of subjects with both AD and diabetes, relative to healthy individuals (reviewed in Duarte, 2016). Accordingly, altered metabolite profiles have been also reported in the brain of diabetes and HFD-induced obesity models (van der Graaf et al., 2004; Duarte et al., 2009; Wang et al., 2012; Lizarbe et al., 2018; Ribeiro et al., 2018).

In the present study, we tested the hypothesis that the severity of the metabolic syndrome is related to the degree of neurochemical alterations, as well as brain function deficits. For that, mice were fed diets containing different amounts of fat during 6 months, which lead to different degrees of glucose intolerance and insulin resistance (Soares et al., 2018). We then evaluated spatial memory performance, metabolic profiles of the hippocampus, cortex and hypothalamus, and levels of proteins specific to synaptic and glial compartments.

## Materials and Methods

### Animals

Animal experiments were performed with the approval by the local ethics committee (Service de la Consommation et des Affaires Vétérinaires, Epalinges, Switzerland). Sample size estimation was based on previous MRS experiments (Gapp et al., 2017; Lizarbe et al., 2018). Eleven-week-old male C57BL/6J mice were purchased from Charles River (L’Arbresle, France), and housed in groups of 3–4 on a 12-h light-dark cycle with lights on at 07:00, room temperature at 21–23°C, and humidity at 55–60%. Mice were randomly split in three groups (*n* = 11/group), and dietary interventions started after 1 week of acclimatization to the facility, i.e., at 3 months of age. During 6 months, mice were fed standardized diets containing 10%, 45%, or 60% of kilocalories from lard-based fat (D12450B, D12451, D12492, Research Diets, New Brunswick, NJ, United States), as previously detailed (Soares et al., 2018). Food and tap water were provided *ad libitum*. After this 6-month housing period, the following procedures were carried out sequentially with 2 days of interval: behavior experiments, one MRS scan session, glucose tolerance tests, tissue collection.

### Behavioral Tasks

Exploratory behavior and locomotor activity of mice was evaluated over a 5-min period in the dark, using a squared open field arena measuring 30 cm × 30 cm and 25 cm high, divided in four squares of 15 cm × 15 cm. The number of crossings in these squares and the number of rearing movements with forepaws were quantified as horizontal and vertical activity, respectively. Rearing with the forepaws pressed against the walls was not considered.

Spontaneous alternation was observed in a Y-maze (Duarte et al., 2012a), with each arm of 30 cm long, 5 cm wide, and 25 cm height, converging to an equal angle. Mice were placed at the bottom of one arm in the Y-maze and were allowed to explore freely all three arms for a single 8-min session in the dark. The recorded spontaneous alternation behavior was used to access hippocampal-dependent spatial memory. If the mouse remembers the arm it has just explored, it will then enter the other arm of the maze instead. Complete spontaneous alternation was defined as successive entries into the three arms and expressed as fraction of the possible alternations in the respective test. In additionally to the open field test, the number of entries in the arms of the maze also allowed to access locomotor activity.

### Magnetic Resonance Spectroscopy (MRS)

All experiments were carried out in a 14.1 T magnet with a horizontal bore of 26 cm (Magnex Scientific, Abingdon, United Kingdom), equipped with a 12-cm internal diameter gradient coil insert (400mT/m, 200μs), and interfaced to a DirectDrive console (Agilent Technologies, Palo Alto, CA, United States). Radio frequency transmission and reception were achieved with a home-built quadrature surface coil composed of two geometrically decoupled single-turn loops of 12 mm inner diameter resonating at 600 MHz. Spontaneously breathing mice were anesthetized with 1–1.5% isoflurane (Animalcare Ltd., York, United Kingdom) in a 1:1 O_2_:air mixture, and fixed in a home-built mouse holder with a bite bar and two ear inserts. Body temperature was maintained at 37°C by warm water circulation. Respiration and temperature were continuously monitored using a MR-compatible system (Small Animal Instruments, Inc., Stony Brook, NY, United States). Volumes of interest (VOI) were placed in the dorsal hippocampus (1.2 mm × 2.0 mm × 2.1 mm), cortex (0.8 mm × 4.0 mm × 1.6 mm) or hypothalamus (1.8 mm × 2.7 mm × 1.8 mm) according to anatomical landmarks in T_2_-weighted fast-spin-echo images. Field homogeneity in the VOI was achieved with FAST(EST)MAP (Gruetter and Tkác, 2000). Spectra were acquired using SPECIAL with echo time of 2.8 ms and repetition time of 4 s (Mlynárik et al., 2006). We accumulated 210, 320, and 400 scans for the cortex, hippocampus, and hypothalamus, respectively. The order of scanning the regions was random.

The concentrations of brain metabolites were determined with LCModel (Stephen Provencher Inc., Oakville, ON, Canada), including a Mac spectrum in the database and using the unsuppressed water signal measured from the same VOI as internal reference (Gapp et al., 2017). The following metabolites were included in the analysis: alanine, ascorbate, aspartate, creatine, γ-aminobutyrate (GABA), glutamine, glutamate, glutathione, glycine, glycerophosphorylcholine, glucose, lactate, *myo*-inositol, *N*-acetylaspartate, *N*-acetylaspartylglutamate (NAAG), phosphorylethanolamine, phosphorylcholine, phosphocreatine, *scyllo*-inositol, taurine. The Cramér-Rao lower bound (CRLB) was provided by LCModel as a measure of the reliability of the quantification for each metabolite. In most measured spectra, *scyllo*-inositol was below the detection limit, and was excluded from subsequent data analyses. Alanine in the hypothalamus was also below the detection limit. Remaining metabolites had CRLB below 30%. Given the substantial overlap between phosphorylcholine and glycerophosphorylcholine signals, total choline levels are reported.

Three mice (one on 45% fat diet, two on 60% fat diet) were excluded from the study because of abnormally high glutamine concentration in all brain regions investigated, which suggests the occurrence of congenital portosystemic shunting (Cudalbu et al., 2013).

### Glucose Tolerance Test

After a 6-h fast starting at 07:00, a blood sample was collected to determine glucose and insulin levels. Then mice were given an oral load of glucose (1.5 g/kg), and blood glucose was measured after 15, 30, 60, 90, and 120 min from the tail tip with the Breeze glucometer (Bayer, Zürich, Switzerland).

### Tissue Collection

Mice were briefly anesthetized with isoflurane (2% in air). Glycemia under isoflurane anesthesia was measured using the Ascensia Contour glucometer (Bayer). A blood sample was collected from the descending aorta, and then mice were decapitated. The brain was rapidly removed, and brain regions were dissected in ice-cold phosphate-buffered saline (PBS; in mmol/L: 137 NaCl, 2.7 KCl, 1.5 KH_2_PO_4_, 8.1 Na_2_HPO_4_, pH 7.4). Brain and plasma samples were stored at -80°C until further processing.

### Western Blot

Tissue samples were homogenized with a needle sonicator in lysis buffer [in mmol/L: 150 NaCl, 1 ethylenediaminetetraacetic acid (EDTA), 50 tris(hydroxymethyl)aminomethane (Tris)-HCl, 1% (v/v) Triton X-100, 0.5% (w/v) sodium deoxycholate, 0.5% (w/v) sodium dodecylsulfate (SDS), pH 8.0] containing protease inhibitors (Roche, Switzerland). The homogenate was then maintained in constant agitation for 2 h at 4°C. After centrifugation at 3,000 ×*g* for 10 min at 4°C to remove major debris, the supernatant was saved. Total protein content was determined with the bicinchoninic acid assay (kit from Pierce, Thermo Fisher Scientific, Göteborg, Sweden).

Samples were then diluted in NuPAGE-LDS sample buffer [in mmol/L: 141 Tris-base, 106 Tris-HCl, 50 dithiothreitol, 0.51 EDTA, 0.22 Coomassie Brilliant Blue G-250, 0.175 phenol red; 2% (w/v) lithium dodecylsulfate, 10% glycerol, pH 8.5] and heated for 2 min at 95°C, 2–20 μg of protein were loaded in precast NuPAGE Novex 4–12% polyacrylamide gradient gels (Invitrogen, Thermo Fisher Scientific), along with molecular weight standards (Precision Plus Protein standards from Bio-Rad, Sundbyberg, Sweden), and electrophoresis was carried out at 125 V in a 3-(*N*-morpholino)propanesulfonic acid (MOPS) running buffer (in mmol/L: 50 MOPS, 50 Tris Base, 1 EDTA, 0.1% SDS, pH 7.7).

Proteins were transferred onto 0.45 μm polyvinylidene difluoride membranes (GE Life Sciences, Sweden) in a buffer composed of 192 mmol/L glycine, 25 mmol/L Tris and 20% (v/v) methanol (pH 9.2). The membranes were subsequently blocked for 90 min in 5% (w/v) low-fat milk (VWR, Stockholm, Sweden) solution prepared in Tris-buffered saline (TBS) (in mmol/L: 20 Tris, 137 NaCl, pH 7.46) with 0.1% (v/v) tween-20 (TBS-T), and were then incubated with primary antibodies (Table 1) over night at 4°C. After three 15-min washes in TBS-T containing 1% (w/v) milk, membranes were incubated with the respective secondary antibodies conjugated with horseradish peroxidase (Table 1) for 1 h at room temperature. After the second antibody, the membranes were washed three more times in TBS-T and developed in the ChemiDoc (Bio-Rad) using the SuperSignal West Pico PLUS Chemiluminescent substrate (Thermo Scientific).

**Table 1:** Antibodies employed in the present study.

Antigen	Host	Dilution	Supplier	Molecular weight (kDa)^∗^
**Primary antibodies:**				
Gephyrin	R	1:500	Abcam (#ab181382)	90–95
GFAP	R	1:2,000	Abcam (#ab68428)	49–51
Iba-1	G	1:500	Sigma-Aldrich (#SAB2500041)	15–20
PSD-95	R	1:2,000	Abcam (#ab76115)	90–95
SNAP-25	R	1:5,000	Abcam (#ab109105)	25–26
Synaptophysin	R	1:10,000	Abcam (#ab32127)	34–36
Syntaxin-1	R	1:1,000	Sigma-Aldrich (#S1172)	34–39
Syntaxin-4	R	1:1,000	Abcam (#ab184545)	31–39
vGAT	R	1:1,000	Abcam (#ab42939)	53–55
vGluT1	R	1:2,000	Abcam (#ab180188)	61–62
vGluT2	R	1:1,000	Abcam (#ab216463)	63–65
***HRP-conjugated secondary antibodies:***				
Rabbit IgG	D	1 : 10 000	Abcam (#ab6802)	
Goat IgG	D	1 : 10 000	GeneTex (#GTX232040-01)	

*HRP, horseradish peroxidase; D, donkey; G, goat; R, rabbit. ^∗^Molecular weight range observed in the present study.*

### Biochemical Analyses

Plasma corticosterone and leptin were assayed with ELISA kits from Abcam (Cambridge, United Kingdom). Plasma insulin concentration was determined using the mouse insulin ELISA kit from Mercodia (Uppsala, Sweden).

### Statistical Analysis

All results were analyzed using analysis of variance (ANOVA), followed by independent comparisons with the Fisher’s least significant difference (LSD) test. For the analyses of metabolite concentrations, the three brain regions were analyzed together as repeated measures, and *post hoc* analyses were only performed in case of significant effects of fat or fat-region interaction at *P* < 0.05. Results are presented as mean ± SEM unless otherwise stated.

## Results

**FIGURE 1:**
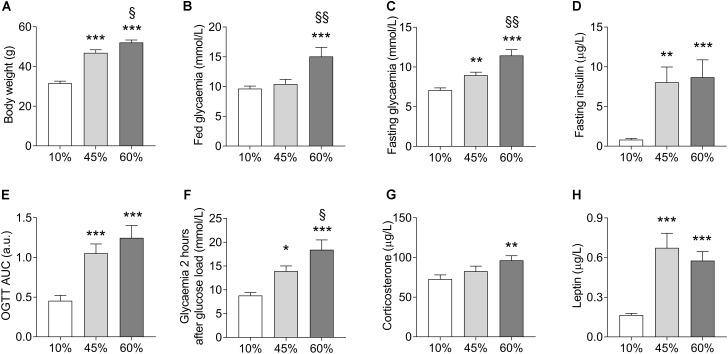
HFD-induced changes on body weight **(A)**, fed glycemia **(B)**, glycemia **(C)**, and plasma insulin **(D)** after a 6-h fasting, area under the curve (AUC) of an oral glucose tolerance test (OGTT) with 1.5 g/kg of glucose **(E)** and glycemia measured 2 h after the glucose load **(F)**, and plasma corticosterone **(G)** and leptin **(H)** concentrations. Data are mean ± SEM. Symbols indicate significant differences in LSD tests after significant ANOVA, comparing HFD fed mice (45% fat or 60%-fat) and controls (10%-fat; ^∗^*P* < 0.05, ^∗∗^*P* < 0.01; ^∗∗∗^*P* < 0.001), or the diets containing 45% and 60% fat (^§^ *P* < 0.05, ^§§^ *P* < 0.01).

Chronic exposure to HFD resulted in development of obesity, mild hyperglycemia, glucose intolerance, and hyperinsulinemia (Figure 1). Weight gain was proportional to the dietary fat content (*F* = 64.2, *P* < 0.001, Figure 1A). Fed glycemia was modified by HFD (*F* = 9.1, *P* = 0.001, Figure 1B), being significantly increased in 60%-fat fed mice compared to controls (+56 ± 14%, *P* < 0.001) and to 45%-fat fed mice (+45 ± 13%, *P* = 0.002). Mice under 10%- and 45%-fat diets showed similar fed glycemia. After fasting for 6 h, mice showed a glycemia that was proportional to the amount of fat in the diet (*F* = 23.1; *P* < 0.001, Figure 1C), while insulinemia was similarly increased in both HFD groups compared to controls (*F* = 9.2, *P* < 0.001, Figure 1D). The diet also had an effect on the fasting glycemia to plasma insulin ratio (*F* = 3.6, *P* = 0.041) that is commonly taken as an indicator of insulin sensitivity. In particular, compared to controls (10%-fat fed mice), the ratio of glucose to insulin was reduced by 94 ± 42% (*P* = 0.034) and 95 ± 42% (*P* = 0.033) in mice from the 45%-fat or 60%-fat groups, respectively. An oral glucose tolerance test after this fasting period revealed an impairment of blood glucose clearance that was also proportional to the amount of fat present in the diet (*F* = 14.6, *P* < 0.001, Figure 1E). This is especially clear when looking at the glycemia at 2 h post the glucose administration (*F* = 13.5, *P* < 0.001, Figure 1F), with 60%-fat fed mice displaying increased glycemia compared to both 45%-fat fed mice (+32 ± 14%, *P* = 0.033) and controls (+109 ± 21%, *P* < 0.001). HFD also affected the circulating concentrations of corticosterone (*F* = 4.2, *P* = 0.025, Figure 1G) and leptin (*F* = 19.0, *P* < 0.001, Figure 1H). In *post hoc* tests, corticosterone was only significantly different between mice under diets with 10% and 60% fat (+32 ± 11%, *P* = 0.007), while leptin was similarly increased in both HFD groups compared to controls.

### Spatial Memory

**FIGURE 2:**
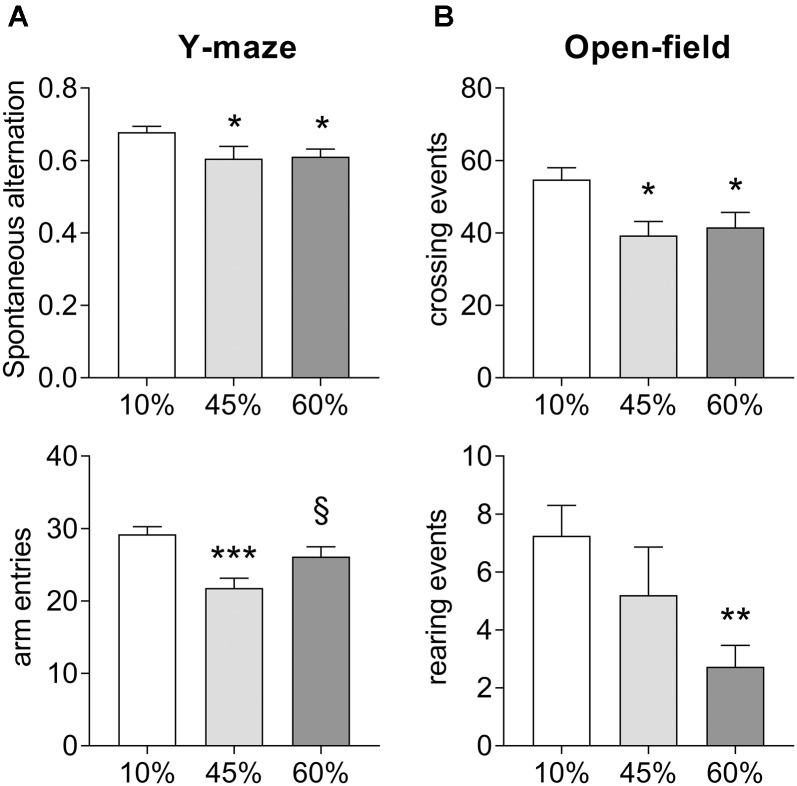
Impact of HFD exposure on the performance in the Y-maze **(A)** and the open-field arena **(B)**. Data are mean ± SEM. Symbols indicate significant differences in LSD tests after significant ANOVA, comparing HDF fed mice (45% fat or 60%-fat) and controls (10%-fat; ^∗^*P* < 0.05, ^∗∗^*P* < 0.01; ^∗∗∗^*P* < 0.001), or the diets containing 45% and 60% fat (^§^ *P* < 0.05).

Exposure to HFD resulted in impaired spatial memory performance, measured as spontaneous alternation in the Y-maze (*F* = 3.8, *P* = 0.030, Figure 2A). In particular, there was a reduction of the Y-maze spontaneous alternation in mice consuming both the 45%-fat diet (-11 ± 5%, *P* = 0.028) and 60%-fat diet (-10 ± 4%, *P* = 0.025), relative to controls. Diet also had an effect on the number of entries in the maze arms (*F* = 8.2, *P* = 0.001), but not resulting in major impairment of exploratory behavior, which was also assessed in the open-field test (Figure 2B). Nevertheless, mice exposed to both 45%-fat and 60%-fat diets showed a reduction in locomotor activity, that is reduced number of crossing events between quadrants of the arena (*F* = 5.5, *P* = 0.008), and reduced vertical explorations of the arena’s space, that is rearing events (*F* = 3.8, *P* = 0.030). Nevertheless, the observed reduction activity did not impair the analysis of spontaneous alteration behavior as it is normalized for the number of possible complete alternations, and there were sufficient arm entries in the Y-maze (Figure 2A).

### Metabolic Profiles

**FIGURE 3:**
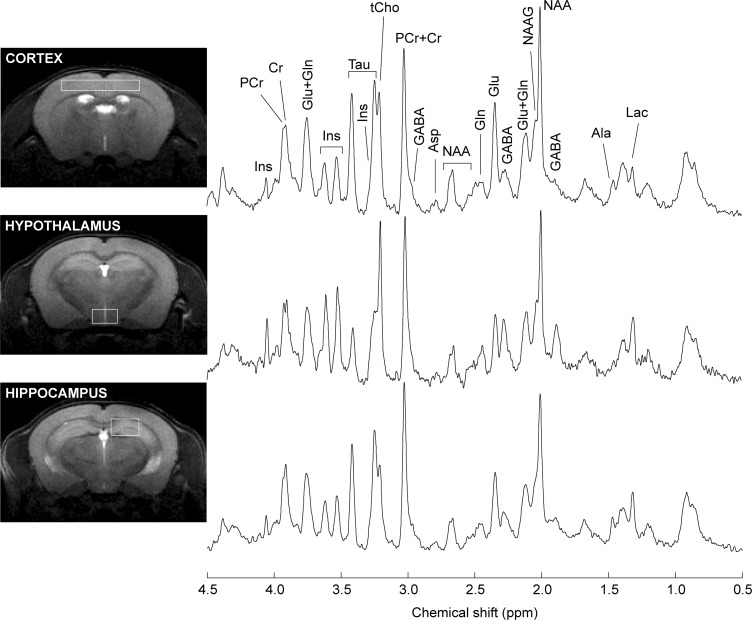
Typical spectra recorded from the mouse hippocampus, cortex and hypothalamus. Spectra were acquired with SPECIAL at 14.1 T. For signal enhancement of the displayed spectra, a Gaussian apodization (gf = 0.08, gfs = 0.02) was applied prior Fourier transformation. Lac, lactate; Ala, alanine; GABA, γ-aminobutyrate; NAA, *N*-acetylaspartate; NAAG, *N*-acetylaspartylglutamate; Glu, glutamate; Gln, glutamine; Asp, aspartate, Cr, creatine; PCr, phosphocreatine; tCho, total choline-containing compounds; Tau, taurine; Ins, *myo*-inositol.

Proton spectra were acquired with an average SNR of 18 ± 3 (standard deviation across all brain areas). Typical spectra are shown in Figure 3. Linewidths at half-height were 11.2 ± 3.0 Hz, 16.2 ± 4.0 Hz and 13.6 ± 2.2 Hz (mean with standard deviation) in the hippocampus, cortex and hypothalamus, respectively.

**FIGURE 4:**
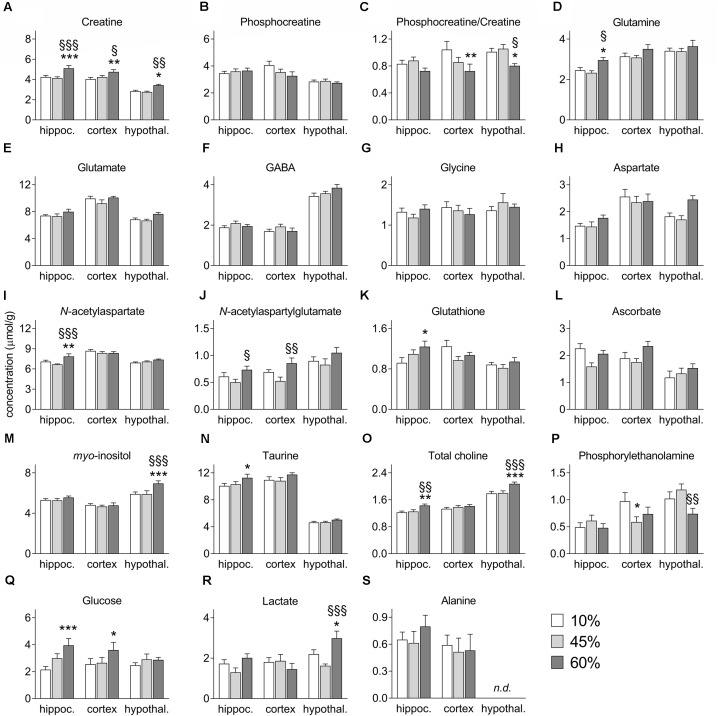
Concentrations of metabolites in the hippocampus (hippoc.), cortex and hypothalamus (hypothal.) of mice fed diets containing 10%, 45%, and 60% fat. Panels **(A–S)** show alterations of each metabolite as presented in the text. Data are mean ± SEM (μmol/g). Symbols indicate significant differences in LSD tests after significant ANOVA, comparing HFD fed mice (45% fat or 60%-fat) and controls (10%-fat; ^∗^*P* < 0.05, ^∗∗^*P* < 0.01; ^∗∗∗^*P* < 0.001), or the diets containing 45% and 60% fat (^§^ *P* < 0.05, ^§§^ *P* < 0.01, ^§§§^ *P* < 0.001).

The amount of fat in the diet significantly impacted the levels of creatine (*F* = 13.7, *P* < 0.001, Figure 4A), which also varied across analyzed brain areas (region effect *F* = 55.1, *P* < 0.001; interaction *F* = 0.9, *P* = 0.506). Although phosphocreatine levels were not impacted by HFD exposure (Figure 4B), the content of fat was associated to reductions of the phosphocreatine-to-creatine ratio (*F* = 13.1, *P* = 0.009, Figure 4C), which suggests impaired energy metabolism. This effect was particularly prominent in the cortex (-31%, *P* = 0.003) and hypothalamus (-20%, *P* = 0.050), and not significant in the hippocampus (-13%, *P* = 0.303) of the 60%-fat group compared to controls.

High-fat diet caused a general increase of glutamine levels (*F* = 7.9, *P* = 0.050, Figure 4D), and there was also an effect of brain region on glutamine levels (region *F* = 33.3, *P* < 0.0001; interaction *F* = 0.9, *P* = 0.717), and *post hoc* analyses revealed a significant increase the hippocampus of mice fed 60%-fat diet relative to controls (+21 ± 10%, *P* = 0.036), as well as relative to mice fed 45%-fat diet (+27 ± 10%, *P* = 0.011). Interestingly glutamate levels were not significantly affected by the diet (Figure 4E). Brain concentrations the neuro-transmitting amino acids GABA (Figure 4F), glycine (Figure 4G), and aspartate (Figure 4H) also remained unaltered by HFD exposure.

The levels of the neuronal marker *N*-acetylaspartate showed a significant diet-region interaction (interaction *F* = 6.7, *P* = 0.006; region *F* = 44.2, *P* < 0.001; fat *F* = 4.5, *P* = 0.066, Figure 4I). *Post hoc* analyses identified an increase of *N*-acetylaspartate concentration in the hippocampus of mice exposed to 60%-fat diet compared to controls (+11 ± 4%, *P* = 0.007), and compared to 45%-fat fed mice (+18 ± 4%, *P* < 0.001).

*N*-Acetylaspartylglutamate levels were modified by fat content in the diet (*F* = 12.8, *P* = 0.015, Figure 4J) and varied across brain areas (*F* = 20.4, *P* < 0.001), without interaction (*F* = 0.5, *P* = 0.918). However, no significant changes were detected in *post hoc* analyses between controls and mice in either HFD group. However, significant differences were observed between 45%-fat and 60%-fat groups in the cortex (+36 ± 22%, *P* = 0.005) and hippocampus (+46 ± 20%, *P* = 0.046).

We found a diet-region interaction effect on the concentration of glutathione (interaction *F* = 9.8, *P* = 0.047; region *F* = 10.9, *P* = 0.006; diet *F* = 2.7, *P* = 0.247, Figure 4K). *Post hoc* analyses showed a significant increase of hippocampal glutathione levels in the 60%-fat group compared to controls (+35 ± 13%, *P* = 0.011). The concentrations of the antioxidant Asc were not significantly modified by HFD exposure (Figure 4L).

There was a significant diet-region interaction effect on the concentration of *myo*-inositol (interaction *F* = 4.9, *P* = 0.029; region *F* = 45.4, *P* < 0.001; diet *F* = 5.2, *P* = 0.071, Figure 4M). *Post hoc* analyses indicated that the increase of *myo*-inositol was exclusive to the hypothalamus (+18 ± 5%, *P* < 0.001 for mice exposed to 60%-fat diet vs. controls; and +18 ± 5%, *P* < 0.001 vs. 45%-fat).

High-fat diet caused an increase of taurine levels (*F* = 1.3, *P* = 0.047, Figure 4N), which also varied across the three brain regions (*F* = 85.9, *P* < 0.001) without diet-region interaction (*F* = 0.2, *P* = 0.788). *Post hoc* analyses showed a significant difference of hippocampal taurine content between the mice exposed 60%-fat diet and controls (+12 ± 0.5%, *P* = 0.022).

The concentration of total choline-containing compounds was affected by HFD exposure (*F* = 6.8, *P* = 0.001, Figure 4O) and varied across brain regions (*F* = 68.1, *P* < 0.001) without interaction between the two factors (*F* = 1.8, *P* = 0.149). The content of total choline was significantly higher in mice exposed to 60%-fat diet in the hippocampus (+17 ± 6%, *P* = 0.007 vs. controls; +14 ± 6%, *P* = 0.018 vs. 45%-fat) and hypothalamus (+16 ± 4%, *P* < 0.001 vs. controls; +15 ± 4%, *P* < 0.001 vs. 45%-fat), but not in the cortex.

A significant diet-region interaction effect was observed on phosphorylethanolamine levels (interaction *F* = 8.7, *P* = 0.022; region *F* = 19.8, *P* < 0.0001; fat *F* = 3.2, *P* = 0.252, Figure 4P). *Post hoc* analyses identified a 40 ± 16% reduction of phosphorylethanolamine concentration in the cortex of mice exposed to 45%-fat diet compared to controls (*P* = 0.017). Such reduction in mice exposed to the 60%-fat diet did not reach statistical significance (-24 ± 17%, *P* = 0.148). The concentration of phosphorylethanolamine in the hypothalamus was different between the two HFD groups (*P* = 0.009).

Brain glucose levels were increased with the amount of fat in the diet (*F* = 12.6, *P* = 0.037, Figure 4Q) with no region (*F* = 0.8, *P* = 0.513) or interaction (*F* = 4.4, *P* = 0.146) effects. Compared to control mice, mice exposed to 60%-fat diet displayed a glucose concentration increase of 84 ± 25% in the hippocampus (*P* < 0.001) and of 41 ± 20% in the cortex (*P* = 0.049), but negligible in the hypothalamus (+16 ± 21%, *P* = 0.442).

We observed a significant diet-region interaction effect on the levels of lactate (interaction *F* = 11.9, *P* = 0.005, region *F* = 10.1, *P* = 0.002, fat *F* = 6.9, *P* = 0.080, Figure 4R). *Post hoc* analyses identified an increase of lactate concentration in the hypothalamus of mice exposed to 60%-fat diet compared to controls (+36 ± 16%, *P* = 0.025), and compared to 45%-fat fed mice (+84 ± 21%, *P* < 0.001). Levels of alanine were not significantly modified by HFD exposure (Figure 4S).

### Synaptic Degeneration

**FIGURE 5:**
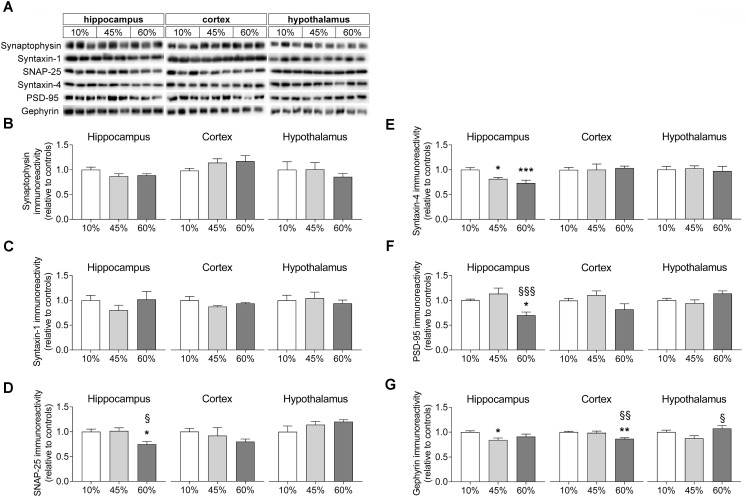
HFD-induced changes on levels of synaptic proteins. **(A)** Shows typical Western blot of synaptophysin **(B)**, syntaxin-1 **(C)**, SNAP-25 **(D)**, syntaxin-4 **(E)**, PSD-95 **(F)**, and gephyrin **(G)**. Samples from three mice of each group were loaded in SDS-PAGE gels (2 μg of protein of syntaxin-1 and synaptophysin, 5 μg of protein for the remaining proteins). Measured immunoreactivity was normalized to the average of the three controls (10%-fat group, white bar) to allow pooling data from different gels. Data are mean ± SEM of *n* = 6. Symbols indicate significant differences in LSD tests after significant ANOVA, comparing HFD fed mice (45% fat or 60%-fat) and controls (10%-fat; ^∗^*P* < 0.05, ^∗∗^*P* < 0.01; ^∗∗∗^*P* < 0.001), or the diets containing 45% and 60% fat (^§^ *P* < 0.05, ^§§^ *P* < 0.01).

In order to infer on synaptic degeneration, we measured the density of proteins located in synapses (Figure 5). Levels of synaptophysin were not significantly impacted by HFD exposure in any of the brain regions investigated (hippocampus *F* = 2.4, *P* = 0.112; cortex *F* = 1.5, *P* = 0.250; hypothalamus *F* = 0.4, *P* = 0.661), although one may notice a tendency for reduced synaptophysin content in the hippocampus of HFD mice relative to controls (*P* = 0.054 and *P* = 0.090 for 45% and 60%-fat versus controls, respectively). Also levels of syntaxin-1 were similar across experimental groups in the hippocampus (*F* = 0.9, *P* = 0.414), cortex (*F* = 1.8, *P* = 0.240) and hypothalamus (*F* = 0.3, *P* = 0.764). The synaptosomal-associated protein SNAP-25 was modified in the hippocampus of HFD-exposed mice (*F* = 6.8, *P* = 0.029), but not in the cortex or hypothalamus. In particular, SNAP-25 in the hippocampus on mice the fed 60%-fat diet was reduced by 25 ± 8% (*P* = 0.021) when compared to controls and by 26 ± 8% (*P* = 0.017) when compared to 45%-fat fed mice. Levels of syntaxin-4, which is another protein of the exocytosis machinery of nerve terminals but mainly occurring in processes of astrocytes (Tao-Cheng et al., 2015), were reduced in the hippocampus (*F* = 9.2, *P* = 0.002) but not cortex (*F* = 0.1, *P* = 0.932) or hypothalamus (*F* = 0.1, *P* = 0.887). In particular, when compared to controls, syntaxin-4 in the hippocampus was reduced by 19 ± 5% (*P* = 0.011) and 27 ± 7% (*P* < 0.001) in mice fed 45%-fat and 60%-fat diets, respectively.

Proteins from the post-synaptic region of synapses were also modified by HFD feeding. Diet impacted the postsynaptic density protein PSD-95 levels in the hippocampus (*F* = 8.1, *P* = 0.002) but not cortex (*F* = 2.9, *P* = 0.076) or hypothalamus (*F* = 3.3, *P* = 0.067). PSD-95 levels were reduced in the hippocampus of mice fed the 60%-fat diet by 30 ± 7% (*P* = 0.012) when compared to controls, and by 38 ± 13% (*P* < 0.001) when compared to mice fed the 45%-fat diet. Levels of gephyrin, another protein from the post-synaptic zone, varied with the amount of fat in the diet in the hippocampus (*F* = 3.8, *P* = 0.044), cortex (*F* = 8.8, *P* = 0.003) and hypothalamus (*F* = 3.9, *F* = 0.035). Compared to controls, the 45%-fat diet caused a significant reduction of gephyrin levels in the hippocampus (-16 ± 5%, *P* = 0.014). In the cortex, 60%-fat diet mice showed reduced gephyrin relative to the other groups (-13 ± 3%, *P* = 0.002 vs. controls; -12 ± 4%, *P* = 0.004 vs. 45%-fat diet). In the hypothalamus there was a reduction of -18 ± 8% in gephyrin levels in mice fed 45%-fat diet versus 60%-fat diet (*P* = 0.011).

**FIGURE 6:**
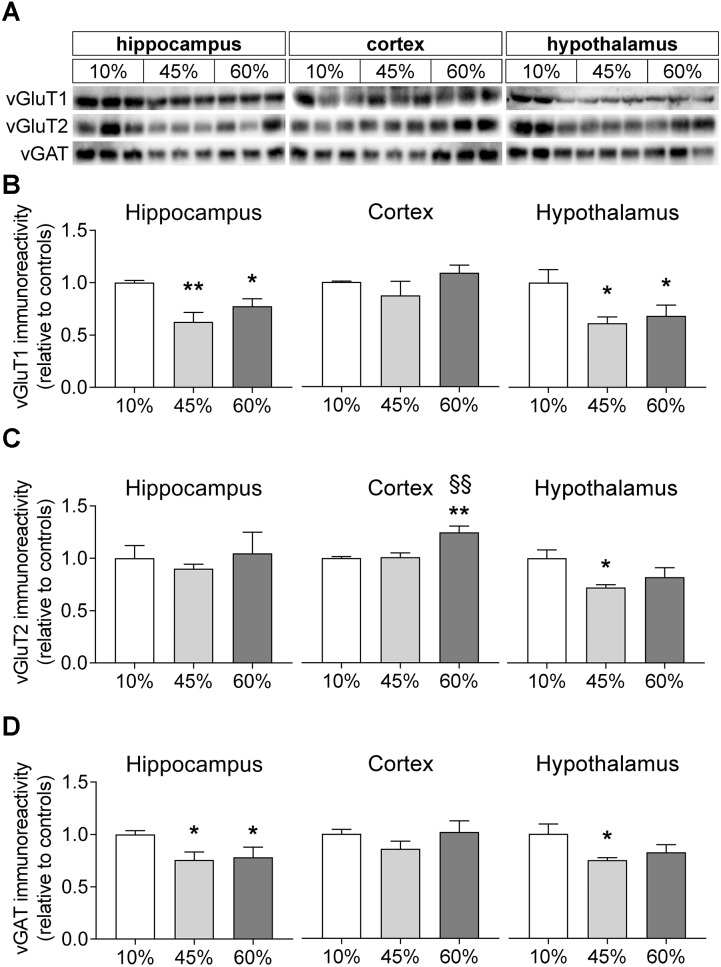
HFD-induced changes on levels of vesicular glutamate and GABA transporters. **(A)** Shows representative Western blots of vGluT1 **(B)**, vGluT2 **(C)**, and vGAT **(D)**. Samples from three mice of each group were loaded in SDS-PAGE gels (20 μg of protein). Measured immunoreactivity was normalized to the average of the three controls (10%-fat group, white bar) to allow pooling data from different gels. Data are mean ± SEM of *n* = 6. Symbols indicate significant differences in LSD tests after significant ANOVA, comparing HFD fed mice (45% fat or 60%-fat) and controls (10%-fat; ^∗^*P* < 0.05, ^∗∗^*P* < 0.01), or the diets containing 45% and 60% fat (^§§^ *P* < 0.01).

We further measured the relative levels of vesicular glutamate and GABA transporters (vGluT1/2 and vGAT; Figure 6). The amount of fat in the diet was associated with a reduction of vGluT1 levels in the mouse hippocampus (*F* = 7.9, *P* = 0.005) and hypothalamus (*F* = 4.3, *P* = 0.033) but not in the cortex (*F* = 1.5, *P* = 0.297). In the hippocampus, both 45%-fat and 60%-fat fed mice showed significantly reduced vGluT1 levels when compared to controls (-38 ± 9%, *P* = 0.001 for 45%-fat diet; -22 ± 7%, *P* = 0.032 for 60%-fat diet). The same was observed in the hypothalamus (-39 ± 14%, *P* = 0.015 for 45%-fat diet; -32 ± 16%, *P* = 0.039 for 60%-fat diet). HFD exposure impacted levels of vGluT2 in the mouse cortex (*F* = 10.3, *P* = 0.001) and hypothalamus (*F* = 3.9, *P* = 0.043) but not in the hippocampus (*F* = 0.3, *P* = 0.748). In the cortex, mice fed 60%-fat diet showed increased cortical vGluT2 relative to the other groups (+25 ± 6%, *P* < 0.001 vs. controls; +24 ± 7%, *P* = 0.001 vs. 45%-fat diet). In the hypothalamus, we observed a significant reduction of vGluT2 levels in mice fed 45%-fat diet (-28 ± 8%, *P* = 0.014 vs. controls). Levels of vGAT were affected by HFD exposure in the hippocampus (*F* = 3.6, *P* = 0.044) and hypothalamus (*F* = 3.6, *P* = 0.049). The hippocampus of mice exposed to both 45%-fat and 60%-fat diet showed less vGAT levels than controls (-25 ± 9%, *P* = 0.024 for 45%-fat diet; -22 ± 10%, *P* = 0.042 for 60%-fat diet). In the hypothalamus, the reduction of vGAT was significant only in mice exposed to 45%-fat diet (-25 ± 10%, *P* = 0.018 vs. controls).

### Gliosis Markers

**FIGURE 7:**
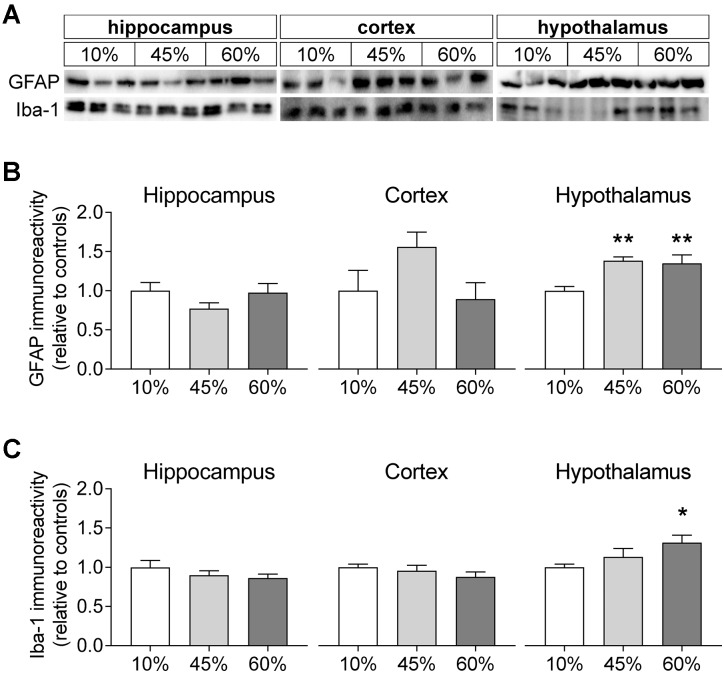
HFD-induced changes on levels of glia-specific proteins. **(A)** Shows typical Western blot of GFAP **(B)** and Iba-1 **(C)**. Samples from three mice of each group were loaded in SDS-PAGE gels (20 μg of protein). Measured immunoreactivity was normalized to the average of the three controls (10%-fat group, white bar) to allow pooling data from different gels. Data are mean ± SEM of *n* = 6. Symbols indicate significant differences in LSD tests after significant ANOVA, comparing HFD fed mice (45% fat or 60%-fat) and controls (10%-fat; ^∗^*P* < 0.05, ^∗∗^*P* < 0.01). No significant differences were observed between the two HFD groups.

To infer on the modification of glial compartments, we measured the levels of glia-specific proteins (Figure 7). The glial fibrillary acidic protein (GFAP) that is specific to astrocytes was modified by fat levels in the diet in the hypothalamus (*F* = 7.8, *P* = 0.005), but not in the hippocampus (*F* = 1.6, *P* = 0.235) and cortex (*F* = 2.6, *P* = 0.155). Relative to controls, the hypothalamus of mice exposed to 45%-fat diet displayed a 38 ± 7% increase of GFAP immunoreactivity (*P* = 0.003), and a similar increase was observed for mice on the 60%-fat diet (+35 ± 12%, *P* = 0.005). Also the ionized calcium-binding adapter molecule 1 (iba-1), a protein present in microglial cells, showed increased levels in the hypothalamus (*F* = 3.5, *P* = 0.045), but not in the hippocampus (*F* = 1.1, *P* = 0.352) and cortex (*F* = 1.1, *P* = 0.360). In particular, there was a 31 ± 10% increase of iba-1 immunoreactivity in the hypothalamus of mice exposed to 60%-fat diet compared to controls (*P* = 0.014).

## Discussion

The present results show that mice chronically exposed to a lard-based HFD develop glucose intolerance associated to reduced insulin sensitivity, impaired hippocampal-dependent spatial memory, and neurochemical alterations in the hippocampus, cortex and hypothalamus. While metabolic profiles of the hippocampus, cortex and hypothalamus were modified in mice fed 60%-fat but not 45%-fat diet, when compared to controls, memory dysfunction was observed in both HFD groups. Accordingly, levels of proteins required for adequate synaptic function were found modified in mice fed diets containing fat at either 45% or 60%, relative to controls. This indicates that synaptic dysfunction may occur in the brain upon diet-induced obesity in the absence of major changes of metabolite concentrations. Nevertheless, this does not exclude the presence of alterations in the rate of certain metabolic pathways.

### Modifications of Energy Metabolism

A general reduction of the phosphocreatine-to-creatine ratio was prominent in 60%-fat fed mice, when compared to the other groups, which suggests a disruption of mechanisms regulating metabolism and maintaining the cellular energy status in the brain. HFD exposure resulted in changes of glucose concentration in the hippocampus and cortex that were parallel to the changes in fed glycemia. However, this was not observed in the hypothalamus, where glucose levels were similar in all diets tested. This suggests that glucose transport and metabolism is differently adapted to the mild hyperglycemia state of 60%-fat fed mice, likely related to the glucose-sensing ability of the hypothalamus. In line with this, the hypothalamus of mice exposed to 60%-fat, but not the hippocampus and cortex, displayed higher levels of lactate than the other experimental groups. The measurement of basal metabolite concentrations, however, does not provide insight into metabolic pathways, namely glycolysis and tricarboxylic acid cycle. By employing ^13^C MRS with ^13^C-labeled glucose we have previously demonstrated that energy metabolism is altered in the hypothalamus upon high-fat feeding (Lizarbe et al., 2018). Modifications of brain energy metabolism were reported in rats fed a fat-rich diet for 3 weeks, namely reduced glucose utilization and increased astrocytic metabolism (Melø et al., 2006). Recently, we also reported that a rat model of lean T2D, the Goto-Kakizaki rat, displays impaired neuronal oxidative metabolism and reduced glutamate-glutamine cycle rate, as well as exacerbated oxidative metabolism in astrocytes (Girault et al., 2018). The relative contributions of fatty acids and glucose as available brain energy substrates, and of neurodegeneration-associated metabolic changes to brain energy dysfunction deserve further investigation. Previous experiments suggest that HFD exposure increases the utilization of fat in detriment of glucose oxidation, at least in the hypothalamus (Lizarbe et al., 2018). This observation, together with the present findings of higher lactate and lower glucose concentrations, leads us to speculate that long-term HFD exposure results in increased glycolysis in for lactate production while other available substrates, such as fats, are metabolized in the tricarboxylic acid cycle.

### Osmolarity Regulation

Changes of the phosphocreatine-to-creatine ratio were due to increases in the creatine concentration rather than decreases of phosphocreatine (see Figures 4A–C), which is in line with the relevance of creatine levels for osmolarity regulation. Osmolarity control in the brain is considered to occur via the concentrations of not only creatine but also taurine and *myo*-inositol, since hyper-osmolarity causes their accumulation (Gullans and Verbalis, 1993). Also *N*-acetylaspartate, which is present in large concentrations in neurons, has been proposed to act as a brain osmolyte (Taylor et al., 1994; Gotoh et al., 1997), and is effectively released to the interstitial space upon osmotic challenges (Sager et al., 1997; Bothwell et al., 2001). Accordingly, taurine and *N*-acetylaspartate in the hippocampus, and *myo*-inositol in the hypothalamus were also increased upon exposure to 60% but not 45% fat in the diet. It should be noted that these modifications in brain osmolytes follow the plasma glucose concentration in the fed state, as well as corticosterone levels, which were significantly higher in 60%-fat fed mice than the other groups. Indeed, in addition to chronic hyperglycemia, it cannot be excluded that glucocorticoid-mediated signaling plays a role in diabetes-induced impaired brain metabolism and function (discussed in Girault et al., 2018).

### Metabolic Indications of Neurodegeneration

*N*-Acetylaspartate reductions are often associated to impaired mitochondrial integrity in neurons, as well as lipid and myelin biosynthesis (Duarte et al., 2012b). Metabolism of *N*-acetylaspartate is compartmentalized between neurons and oligodendrocytes and serves for myelination. *N*-acetylaspartate is mainly stored in neurons, being synthesized from mitochondrial acetyl-CoA and Asp (Baslow, 2003), and its de-acetylation occurs in oligodendrocytes (Kirmani et al., 2002), where it can replenish both acetyl-CoA and oxaloacetate (through aspartate) pools providing carbon skeletons for both energy production and local lipid synthesis, required in the myelination process. In line with this role of *N*-acetylaspartate, Canavan disease is characterized by accumulation of *N*-acetylaspartate due to impaired de-acetylation in oligodendrocytes, which is associated to defects in myelin synthesis and deposition (Chakraborty et al., 2001). Indeed, it was recently suggested that high-fat feeding has an impact on myelination, namely by promoting the loss of oligodendrocyte progenitor cells and mature oligodendrocytes (Yoon et al., 2016).

A significant increase of hippocampal glutathione levels was observed in the 60%-fat group relative to controls, while there was a tendency for reduced glutathione levels in the cortex. It should be noted that the levels of glutathione measured by MRS include both reduced and oxidized forms, which are not distinguishable. Although lower ratio of reduced-to-oxidized glutathione has been reported in short-term HFD feeding (e.g., Alzoubi et al., 2018), no change of brain levels of oxidized and reduced glutathione, or their ratio upon long-term HFD exposure (Li et al., 2013). It should be noted however that Li et al. (2013) reported a non-significant increase of 15–20% of hippocampal reduced glutathione in high- versus low-fat-fed mice, in line with the present observation. It is likely that a total glutathione increase in the hippocampus might reflect a compensatory mechanism to oxidative stress upon chronic HFD exposure. In fact, the brain of HFD-fed mice displays impaired redox homeostasis, including: (1) enhanced activity of enzymes that generate free radicals, such as xanthine oxidase or NADPH oxidase; (2) reduced activity of enzymes with antioxidant action, namely glutathione peroxidase and catalase; (3) increased concentration of free radicals; (4) and oxidative damage to lipids and proteins (e.g., Morrison et al., 2010; Li et al., 2013; Charradi et al., 2017; Liu et al., 2017; Alzoubi et al., 2018; Maciejczyk et al., 2018). Accordingly, the hippocampus of mice fed a 60%-fat diet for 4 months also shows impaired signaling mediated by the transcription factor NRF2 which protects against brain oxidative damage (Morrison et al., 2010). Namely, Morrison et al. (2010) reported HFD-induced reductions of NRF2 protein levels, NRF2-DNA binding activity, and levels of the NRF2 responsive proteins in the hippocampus.

Interestingly, impaired cellular redox regulation in the brain has been proposed to impact oligodendrocyte proliferation (consistent with increased levels of *N*-acetylaspartate, discussed above), which has implications on the maintenance of myelin sheets around axons, and thus brain connectivity (see Corcoba et al., 2016 and references there in).

The content of total choline was significantly higher in hippocampus and hypothalamus, but not cortex. The main contributors to the choline peaks in MRS are the water-soluble glycerophosphorylcholine and phosphorylcholine, which are involved in membrane lipid metabolism (Duarte et al., 2012b). Namely, it is precursor of phosphatidylcholine and, in turn, of sphingomyelin, which is necessary for adequate myelination of axons (Oshida et al., 2003), and is implicated in immune responses (Li et al., 2015). In contrast to phosphorylcholine, a HDF-induced reduction of phosphorylethanolamine concentration was observed in the cortex. It is interesting to note that besides these changes in the concentration of phosphorylethanolamine in the cortex, no other significant metabolic alterations were observed between mice fed the diet containing 45% of fat and those in the control group. Altogether, these results point to alterations on phospholipid metabolism with possible implications on plasma membrane permeability, maintenance of action potentials and conduction of electric impulses, and on events that involve membrane deformation, fusion and fission, such as transcellular signaling, vesicular release of neurotransmitters, gliotransmitters or neuromodulators, mitochondrial dynamics, and autophagy (Aureli et al., 2015; Krols et al., 2016; Lauwers et al., 2016; van Echten-Deckert and Alam, 2018). In addition, lipid metabolism is important for membrane reorganization of astrocytes and microglia in neuroinflammation, which have been proposed to be reflected on MRS-detected changes of choline-containing compounds (discussed in Duarte et al., 2012b). In our study, total choline increased with HFD in the hippocampus and hypothalamus, while increased levels of GFAP and Iba-1, which are signs of inflammation, were exclusively observed in the hypothalamus. These results support the notion that MRS markers do not distinguish neuroinflammation from neurodegeneration and other processes (Zahr et al., 2014; Pardon et al., 2016).

Taurine is an amino acid that, although it is present at 1 μmol/g in the human brain, it reaches relatively large concentrations in the rodent brain (above 5 μmol/g in rats and above 8 μmol/g in mice; Duarte, 2016), thus playing a major role as osmolyte (Duarte et al., 2009 and references therein). Taurine is released from both neurons and glial cells (Hada et al., 1998), and acts as an agonist at receptors of the GABAergic and glycinergic neurotransmission systems (Albrecht and Schousboe, 2005). In the present study, HFD exposure caused a reduction of proteins associated to inhibitory neurotransmission (gephyrin and vGAT), suggesting that increased taurine might be a compensatory mechanism for the loss of inhibitory tone. Moreover, taurine is transported into the mitochondrial matrix where it buffers pH to the optimal value for isocitrate dehydrogenase, which is one of the key enzymes of the tricarboxylic acid cycle regulating energy metabolism and oxidative phosphorylation, contributes to stabilize the pH gradient across the inner-membrane, and thus helps preserving mitochondrial function and preventing oxidative damage (Hansen et al., 2010). Therefore, this HFD-induced increase of taurine levels in the is likely a beneficial adaptation of the brain.

### Microgliosis and Astrogliosis

Increased levels of *myo*-inositol have been suggested to represent astrogliosis in the neurodegeneration-associated inflammatory process (discussed in Duarte et al., 2012b). The HFD-associated increased *myo*-inositol of the present study was exclusive to the hypothalamus. Interestingly, the hypothalamus was also the sole brain region to show significantly HFD-associated increased levels of glial specific proteins, namely GFAP and Iba-1 that are present in astrocytes and microglia, respectively. This overexpression of GFAP and Iba-1 is thus indicative of HFD-associated hyper-reactivity of astrocytes (astrogliosis) and microglia (microgliosis), as we reported recently (Lizarbe et al., 2018). Brain disorders are accompanied by neuroinflammation with astrogliosis and microgliosis generally in response to damage of nearby neuronal processes, but gliosis might become deleterious for the neurons upon chronicity (López-Valdés and Martínez-Coria, 2016). A transient increase in levels of pro-inflammatory cytokines was observed in the hypothalamus within 1–3 days of HFD exposure (e.g., Thaler et al., 2012; Waise et al., 2015), which might constitute a protective or reparative inflammatory process. In contrast, the hypothalamic gliosis in chronic HFD exposure (present study) is likely neurotoxic and has been suggested to participate in the genesis of obesity-associated diabetes since it may cause alterations of food intake, energy expenditure, insulin secretion, hepatic glucose production and metabolism of glucose and fats (Rahman et al., 2018).

The hippocampus and cortex of HFD-exposed mice did not show signs of gliosis, when compared to controls (no significant increase in levels of *myo*-inositol, GFAP or Iba-1). Despite the general consensus that neuroinflammation and astrogliosis occur in insulin resistance and T2D (Saravia et al., 2002; Baydas et al., 2003; Duarte et al., 2009, 2012a; Calvo-Ochoa et al., 2014), conflicting results have been reported regarding astrogliosis in memory-related brain areas upon HFD exposure. This is likely due to the distinct study designs, diet compositions, fat origin, age and/or species. Recent studies do not find HFD-induced increased levels of GFAP in the rat hippocampus (Raider et al., 2016; Ribeiro et al., 2018). In contrast, Tarantini et al. found that mice exposed to a 60%-fat diet from 3 to 8 months of age show mild behavior deficits and neuroinflammation, which are exacerbated by exposure to a cellular stress induced by deletion of the transcription factor *nrf2* that regulates the expression of antioxidant proteins, thus protecting against oxidative damage (Tarantini et al., 2018). A particularly interesting study by Tsai et al. (2018) found that GFAP levels increase in the hippocampus of mice exposed to 60%-fat diet for 3 months, relative to controls, and that HFD consumption leads to a reduction of the length and ramification of astrocytic processes in the CA1 and CA3 regions but not the dentate gyrus of the hippocampus. This retraction of astrocytic processes is of importance for the metabolic support that astrocytes should provide to neurons and their synapses (Sonnay et al., 2017), and in fact Tsai et al. (2018) also reported that HFD reduced levels of the astrocytic glutamate carriers GLT-1 and GLAST in the hippocampus, without changes of glutamine synthase levels. Soontornniyomkij et al. (2016) also found that 5 months of HFD exposure does not change levels of glutamine synthetase in the hippocampus of 5-month-old mice, although HFD increases the density of this protein in aged mice. Altogether, these observations suggest impaired glutamate-glutamine cycle rate that, in turn, may result in glutamatergic dysfunction and consequent accumulation of glutamine in astrocytes. Consistent with impaired glutamate release, we observed reduced levels of vesicular glutamate transporters, mainly vGluT1. However, care should be taken when interpreting changes on levels of carriers and enzymes because they may not reflect changes of their activity in the brain *in vivo*.

Moreover, as mentioned above, observations from different diet-induced obesity studies should be compared with caution. For example, in contrast to the reduced levels of glutamate carriers caused by a 60% HFD (Tsai et al., 2018), hippocampal slices prepared from mice fed a 45%-fat diet for only 2 months displayed enhanced glutamate uptake, increased density of glutamate carriers and reduced levels of the glutamate degrading enzymes glutamine synthetase and GABA-decarboxylase, compared to slices from controls (Valladolid-Acebes et al., 2012). Based on our results, we believe that the exposure to diets containing fat at 45% or 60% elicits different degenerative processes rather than resulting from a simple dose-response phenotype.

Glutamine synthesis takes place in astrocytes (Sonnay et al., 2017). In our study, HFD caused indeed an increase of glutamine levels in the 60%-fat group (but not the 45%-fat) relative to controls, which was particularly significant in the hippocampus. In line with our observations, increased hippocampal glutamine-to-creatine ratio was recently reported in Wistar rats exposed to a 60%-diet from 8 to 12 months of age (Ribeiro et al., 2018). In young adult Fisher 344 rats under a 60%-fat diet for 5 months, Raider et al. (2016) also observed a tendency for higher hippocampal glutamine concentration, and a significantly increased glutamine-to-glutamate ratio, relative to controls. This study, however, reported lower levels of *myo*-inositol and creatine in high versus low fat-fed rats (Raider et al., 2016), contrasting to the findings of the present study (discussed above).

### Dysfunction in Excitatory and Inhibitory Synapses

*N*-Acetylaspartylglutamate (NAAG) levels increased in the cortex and hippocampus with HFD exposure. Similar NAAG changes were observed in HFD-exposed rats (Raider et al., 2016). NAAG is synthetized from ATP-dependent condensation of *N*-acetylaspartate and glutamate in neurons, packed into synaptic vesicles of presynaptic terminals, including those of pyramidal neurons in the cortex and limbic system, is co-released with glutamate, and acts as an antagonist of the glycine site of the NMDA receptor (Duarte and Xin, 2018 and referenced therein). Importantly, NAAG also acts on the metabotropic type II glutamate receptor mGluR3 inhibiting the release of neurotransmitters, such as glutamate, GABA and glycine (Neale, 2011). In HFD-exposed mice, increased NAAG levels would thus reduce glutamatergic neurotransmission by both inhibiting the activity of NMDA receptors and augmenting the negative feedback of glutamate release through its agonist action on presynaptic mGluR3 receptors. High NAAG concentration might prevent glutamate excitotoxicity, which is important when astrocytes have impaired glutamate clearance (see above), but on the other hand sufficient glutamatergic activity is necessary for proper function, including memory performance.

Consistent with reduced rate of the glutamate-glutamine cycle and with synaptic dysfunction in general, we observed a reduction in the levels of some pre- and post-synaptic proteins and of vesicular glutamate and GABA transporters in mice exposed to diets containing either 45% or 60%, relative to controls. We measured the density of two presynaptic proteins that are target SNARE (Soluble *N*-ethylmaleimide-sensitive factor Attachment protein REceptor) proteins required for vesicle fusion, namely syntaxin-1 and SNAP-25 (Söllner et al., 1993), and the density of synaptophysin that is a major component of presynaptic vesicles (Valtorta et al., 2004). SNAP-25 was reduced in the hippocampus of mice fed 60%-fat diet, when compared to controls, and there was also a tendency for hippocampal synaptophysin levels to be reduced with increasing fat content in the diet. Similar results were found for the density of vGluT1 and vGAT in the hippocampus, which suggest a reduction of the number of excitatory and inhibitory synaptic vesicles that are available for neurotransmission. Deficits in gliotransmission cannot be excluded since vesicular transporters are present in both neurons and astrocytes (see Schubert et al., 2011 and references therein). Therefore, we further measured the levels of syntaxin-4. While syntaxin-1 is mostly located in neurons, namely in the plasma membrane of the presynaptic terminal, syntaxin-4 is particularly concentrated in glial cells, namely in peri-synaptic astrocytic processes (Schubert et al., 2011; Tao-Cheng et al., 2015). In fact, the level of syntaxin-4 was also found reduced in the hippocampus of mice from either HFD group, confirming also a dysfunction at the level of gliotransmission. We further measured the density of PSD-95 that is associated to excitatory postsynaptic densities (Hunt et al., 1996), and gephyrin that is present at the inhibitory postsynaptic density, where it interacts with glycine and γ-aminobutyric acid type A (GABA_A_) receptors (Maric et al., 2011). In the hippocampus, HFD also led to a reduction of PSD-95 (60%-fat group only) and of gephyrin (only significant for 45%-fat), compared to controls. Reduced level PSD-95 in the hippocampus of 45%-fat fed mice (for 2 weeks) relative to controls was also reported previously (Arnold et al., 2014). This synaptic dysfunction might be an important contributor for the hippocampal-dependent spatial memory impairment that was measured through the reduced spontaneous alternation in the Y-maze. In the cortex, only the levels of gephyrin were reduced in 60% fat fed mice versus controls, suggesting reduced inhibitory tone by the GABAergic system. In the hypothalamus, only vesicular neurotransmitter transporters and not the remaining measured synaptic proteins were reduced by HFD exposure, suggesting reduced neurotransmission capacity in both orexigenic and anorexigenic neurons (Meister, 2007; Moraes et al., 2009).

## Conclusion

In sum, increasing the dietary amount of lard-based fat from 10 to 45% or 60% of the total energy intake leads to obesity accompanied by increased fasting blood glucose and insulin (pointing toward reduced insulin sensitivity), reduced glucose tolerance and increased plasma leptin. However, compared to controls (10%-fat) increased fed glycemia and plasma corticosterone were only observed in the 60%-fat fed mice. Exposure to both 45%-fat and 60%-fat diets resulted in deterioration of systems involved in neuro- and gliotransmission in the hippocampus, and in impaired spatial memory performance. Metabolic alterations measured by ^1^H MRS were generally observed in mice exposed to 60%-fat but not 45%-fat diet. Therefore, we conclude that different features of the metabolic syndrome result in distinct neurochemical alterations in the brain, all likely contributing for memory impairment. Moreover, chronically increased blood glucose and/or corticosterone appear to be main drivers for changes in brain metabolite levels.

## Author Contributions

JD designed the study and wrote the manuscript. All authors performed the experiments, analyzed the data, and revised the manuscript.

## Conflict of Interest Statement

The authors declare that the research was conducted in the absence of any commercial or financial relationships that could be construed as a potential conflict of interest.

## Abbreviations

Aβ: amyloid β
AD: Alzheimer’s disease
ANOVA: analysis of variance
CRLB: Cramér-Rao lower bound
GABA: γ-aminobutyrate
HFD: high-fat diet
IGF-1: insulin-like growth factor 1
LSD: least significant difference
MOPS: 3-(*N*-morpholino)propanesulfonic acid
MRS: magnetic resonance spectroscopy
NAAG: *N*-acetylaspartylglutamate
TBS: Tris-buffered saline
T2D: type 2 diabetes
VOI: volume of interest
